# Calpain-10 regulates actin dynamics by proteolysis of microtubule-associated protein 1B

**DOI:** 10.1038/s41598-018-35204-x

**Published:** 2018-11-13

**Authors:** Tomohisa Hatta, Shun-ichiro Iemura, Tomokazu Ohishi, Hiroshi Nakayama, Hiroyuki Seimiya, Takao Yasuda, Katsumi Iizuka, Mitsunori Fukuda, Jun Takeda, Tohru Natsume, Yukio Horikawa

**Affiliations:** 1 Molecular Profiling Research Center for Drug Discovery (molprof), National Institute of Advanced Industrial Science and Technology (AIST), 2-3-26 Aomi, Koto-ku, Tokyo, 1345-0064 Japan; 2 Division of Molecular Biotherapy, Cancer Chemotherapy Center, Japanese Foundation for Cancer Research, 3-8-31, Ariake, Koto-ku, Tokyo, 135-8550 Japan; 3 Biomolecular Characterization Team, RIKEN Advanced Science Institute, 2-1 Hirosawa, Saitama, 351-0198 Japan; 4 Laboratory of Membrane Trafficking Mechanisms, Department of Developmental Biology and Neurosciences, Graduate School of Life Sciences, Tohoku University, Aobayama, Aoba-ku, Sendai, Miyagi 980-8578 Japan; 5 Department of Diabetes and Endocrinology, Gifu University School of Medicine, 1-1 Yanagido, Gifu, 501-1194 Japan; 6 Present Address: Fukushima Medical University, 1 Hikariga-oka, Fukushima, 960-1295 Japan

## Abstract

Calpain-10 (CAPN10) is the calpain family protease identified as the first candidate susceptibility gene for type 2 diabetes mellitus (T2DM). However, the detailed molecular mechanism has not yet been elucidated. Here we report that CAPN10 processes microtubule associated protein 1 (MAP1) family proteins into heavy and light chains and regulates their binding activities to microtubules and actin filaments. Immunofluorescent analysis of Capn10^−/−^ mouse embryonic fibroblasts shows that MAP1B, a member of the MAP1 family of proteins, is localized at actin filaments rather than at microtubules. Furthermore, fluorescence recovery after photo-bleaching analysis shows that calpain-10 regulates actin dynamics via MAP1B cleavage. Moreover, in pancreatic islets from CAPN10 knockout mice, insulin secretion was significantly increased both at the high and low glucose levels. These findings indicate that deficiency of calpain-10 expression may affect insulin secretion by abnormal actin reorganization, coordination and dynamics through MAP1 family processing.

## Introduction

Calpains are a family of intracellular non-lysosomal calcium-activated neutral cysteine proteases known to cleave various substrate proteins and modulate their activities. There are 16 calpains, some of which are ubiquitously expressed and others displaying tissue-specific distribution. Some calpains contain a penta-EF-hand domain (typical or classical calpains), and others do not (atypical calpains). Several calpain family members are implicated in the development of various diseases including Alzheimer’s disease, cataracts, ischemic stroke, traumatic brain injury, limb-girdle muscular dystrophy 2A and type 2 diabetes mellitus (T2DM)^1^. Calpain-10 (CAPN10) is a member of the atypical calpain group, and was identified as the first susceptibility gene for T2DM^2^. The development of T2DM involves reduced insulin secretion that cannot compensate for insufficient insulin action. Genes that affect insulin secretion are therefore potential susceptibility genes for type 2 diabetes.

Several reports indicate that CAPN10 is implicated in insulin secretion and action. It has been reported that CAPN10 facilitates glucose transporter 4 (GLUT4) translocation. Reduced CAPN10 expression has been found to decrease insulin-stimulated GLUT4 vesicle translocation, actin reorganization and glucose uptake in adipocytes^3^. It was also reported that targeted suppression of CAPN10 expression impairs insulin-stimulated glucose uptake in skeletal muscle^4^. Moreover, inhibition of calpain activities using calpain inhibitor, although not targeted suppression, was shown to impair actin reorganization and insulin secretion from beta-cells^5^. Enhancement of insulin secretion by short-term exposure to calpain inhibitors is mediated by accelerated exocytosis of insulin granules^6^, whereas a 48 h long-term exposure of mouse islets to calpain inhibitors suppresses glucose-stimulated insulin secretion^7^.

Thus, previous reports show that CAPN10 is implicated in insulin secretion and action and facilitates actin reorganization during glucose-stimulated insulin secretion and insulin-stimulated glucose uptake. However, the molecular mechanisms and substrate proteins of CAPN10 have not been elucidated. Since CAPN10 is expressed in various tissues, target substrates of the protein and other related factors could act together in development of diabetes. In this study using a proteomic approach, we demonstrate that microtubule-associated protein 1 (MAP1) family proteins are the substrate proteins of CAPN10 activity. Our findings show that CAPN10 is required in processing MAP1 family proteins and regulation of their binding activities to microtubules and actin, both of which could readily affect insulin secretion and action through impaired actin reorganization, coordination and dynamics.

## Results

### Calpain-10 cleaves MAP1 family proteins

**Figure 1:**
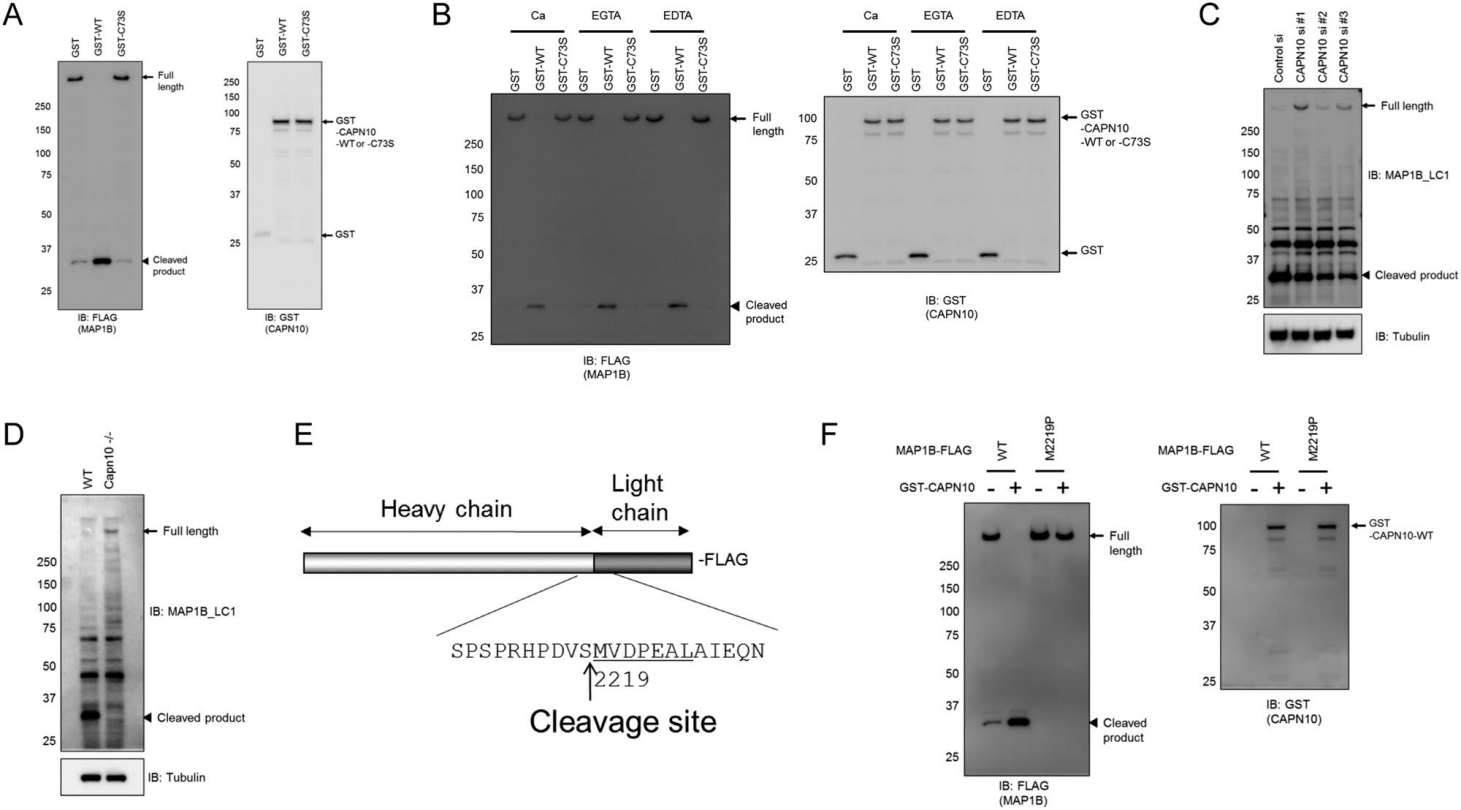
CAPN10 cleaves MAP1B. (**A**) Western blot analysis of cell lysates expressing GST-CAPN10 with MAP1B-FLAG. Full-length uncleaved MAP1B, cleavage product of MAP1B and CAPN10 bands are indicated by arrows, arrowheads and asterisks, respectively. (**B**) MAP1B-FLAG was incubated with GST-CAPN10 in the presence of Ca^2+^ or EGTA *in vitro*. WT and C73S indicate CAPN10 and −C73S mutant. (**C**) Western blot of cell lysates from HEK293T cells transiently transfected CAPN10 siRNA (CAPN10 si #1 or CAPN10 si #2) or control siRNA (control si). (**D**) Cell lysates from wild-type (WT) and CAPN10 knock-out (Capn10^−/−^) mouse embryonic fibroblasts. (**C** and **D**) were analyzed by western blotting and probed with anti-MAP1B-C-terminus (C-term) and tubulin as a loading control. (**E**) Schematic representation of MAP1B denotes the site of cleavage after serine 2218. (**F**) MAP1B-FLAG (WT) or MAP1B-M2219P-FLAG (M2219P) was co-transfected with control plasmid (−) or GST-CAPN10 (+). Cell lysates from transfected HEK293T cells were analyzed by western blotting and probed with anti-FLAG antibody. Full-length uncleaved MAP1B, cleavage product of MAP1B and CAPN10 bands are indicated by arrows, arrowheads and arrows, respectively.

To identify the substrate proteins for CAPN10, we employed a proteomic approach. FLAG-tagged CAPN10 was expressed in HEK293T cells as the bait protein and pulled down with FLAG M2 beads. Proteins co-precipitated with FLAG-tagged CAPN10 were eluted from FLAG M2 beads and the eluted fraction was analyzed by the nano-LC MS/MS system^8^. Because the co-precipitated proteins detected by MS were candidate calpain-10 substrates, we co-transfected each of the proteins with calpain-10 in HEK293T cells. Each expressed candidate protein was then analyzed by western blotting to determine whether or not it became cleaved. Among these candidate proteins, only microtubule-associated protein 1B (MAP1B) was degraded or processed when CAPN10 was co-transfected. The full-length protein of MAP1B (~300 KDa) was not observed and only smaller bands (34 KDa) were detected (Fig. 1A). A full-length band was clearly observed in cells co-transfected with control plasmid (Fig. 1A).

To confirm that the observed MAP1B cleavage was due to the cysteine protease activity of CAPN10, we co-transfected C73S mutant (substituted Cys73 by Ser) with MAP1B, as Cys73 is a conserved cysteine residue at the putative catalytic center of CAPN10 predicted from primary sequence alignment of other calpain members. In C73S mutant co-transfected cells, the full-length MAP1B protein was observed, and was similar to that in control transfection (Fig. 1A). These results indicate that MAP1B is cleaved by proteolytic activity of CAPN10. While there are 15 calpain members, none of the other calpains except CAPN3 was found to cleave MAP1B in the co-transfection experiments described above (Fig. S1A). Tissue specific CAPN3 may also cleave MAPs at a site(s) different from CAPN10. CAPN10 also cleaved the other MAP1 family members, MAP1A and MAP1S (Fig. S1B,C), demonstrating that all MAP1 family members are specifically cleaved by CAPN10.

To further confirm that MAP1B is cleaved by CAPN10 *in vivo*, we prepared both recombinant proteins and performed *in vitro* digestion assay. Wild-type CAPN10 cleaved MAP1B *in vitro*, while C73S did not (Fig. 1B). We further examined the proteolytic activity to determine whether Ca^2+^ is involved, since CAPN10 is an atypical calpain member lacking the penta-EF-hand calcium-binding motif, which is well conserved among typical calpain members. *In vitro* assay showed that CAPN10 exhibited proteolytic activity *in vitro* by cleaving MAP1B with and without Ca^2+^ (Fig. 1B). CAPN10 still was capable of cleaving MAP1B in the presence of 5 mM EDTA, indicating that CAPN10 does not require Ca^2+^ or other metal ions for its proteolytic activity.

We then performed siRNA-based knockdown experiment to assess whether endogenous CAPN10 cleaves MAP1B protein in HEK293T cells. Western blot analysis with an antibody against the MAP1B C-terminus detected no full-length form of MAP1B and a substantial amount of low molecular weight C-terminal cleavage product (~34 KDa). This finding indicates that endogenous MAP1B was fully processed in the cells treated with control siRNA (Fig. 1C). Three independent siRNAs apparently reduced a low molecular weight cleavage product (~34 KDa), while uncleaved MAP1B (~300 KDa) was clearly observed. To further confirm endogenous MAP1B cleavage, we analyzed mouse embryonic fibroblast (MEF) cells from WT and calpain-10 knockout (Capn10^−/−^) mice (Fig. 1D). Western blot analysis showed only full-length MAP1B in Capn10^−/−^ MEF cells. In contrast, the full-length MAP1B band was not detected, while only the cleaved band was observed in WT MEF, similar to HEK293 cells treated with control siRNA. Both MAP1A and 1S were cleaved by CAPN10 in the same manner as MAP1B (Fig. S2A–D). Taken together, this series of experiments indicates that MAP1 family members could be direct substrates of CAPN10 both *in vitro* and *in vivo*.

### CAPN10 is a MAP1B-processing enzyme in the microtubule binding activity of MAP1B

MAP1 family proteins are known to be synthesized a**s** latent single, large precursor proteins, and then processed by proteolytic cleavage near the carboxyl terminus. This leads to the generation, of heavy chain (HC: 300 KDa) and light chain (LC: 34 KDa) heterodimer formation. However, the precise processing site of MAP1B has not been determined and the processing protease for MAP1 family proteins has not been identified.

We therefore investigated whether CAPN10 processes MAP1B to generate the LC and HC of MAP1B. We first incubated purified proteins of MAP1B, CAPN10, and CAPN10-C73S (Fig. S3) and the reaction mixtures were then separated by SDS-PAGE and transferred to a PVDF membrane. The 34 KDa bands, which were more intensely stained in the CAPN10 mixture than in the C73S mixture, were subjected to N-terminal amino acid sequencing (Fig. S4). This experiment showed that the first seven N-terminal amino acid sequence was MVDPEAL, clearly demonstrating that the N-terminal amino acid residue is Met2219 (Fig. 1E). This result is consistent with a previous study that narrowed down the processing site of MAP1B (predicted site is between 2216–2221 Accession number P46821) by combined cyanogen bromide and acid cleavage as well as N-terminal sequencing^9^.

To further confirm the Ser2218-Met2219 peptide bond of MAP1B as the processing site of CAPN10 *in vivo*, Met2219 of MAP1B was replaced by a Pro residue (M2219P). M2219P was co-transfected with CAPN10 in HEK293T cells and analyzed by western blotting. We observed that CAPN10 did not process M2219P, and the 34 KDa band seen in MAP1B co-transfection was not detected (Fig. 1F). We then introduced the same mutation into MAP1A and MAP1S by sequence alignment and performed a cleavage assay. As expected, single point mutants of MAP1A (M2579P) and MAP1S (M842P) were not processed by CAPN10 (Fig. S5A–C). These data indicate that CAPN10 is the processing enzyme that generates HCs and LCs of MAP1 family members.

### The microtubule-binding domain of LC is activated by CAPN10-mediated proteolytic processing

**Figure 2:**
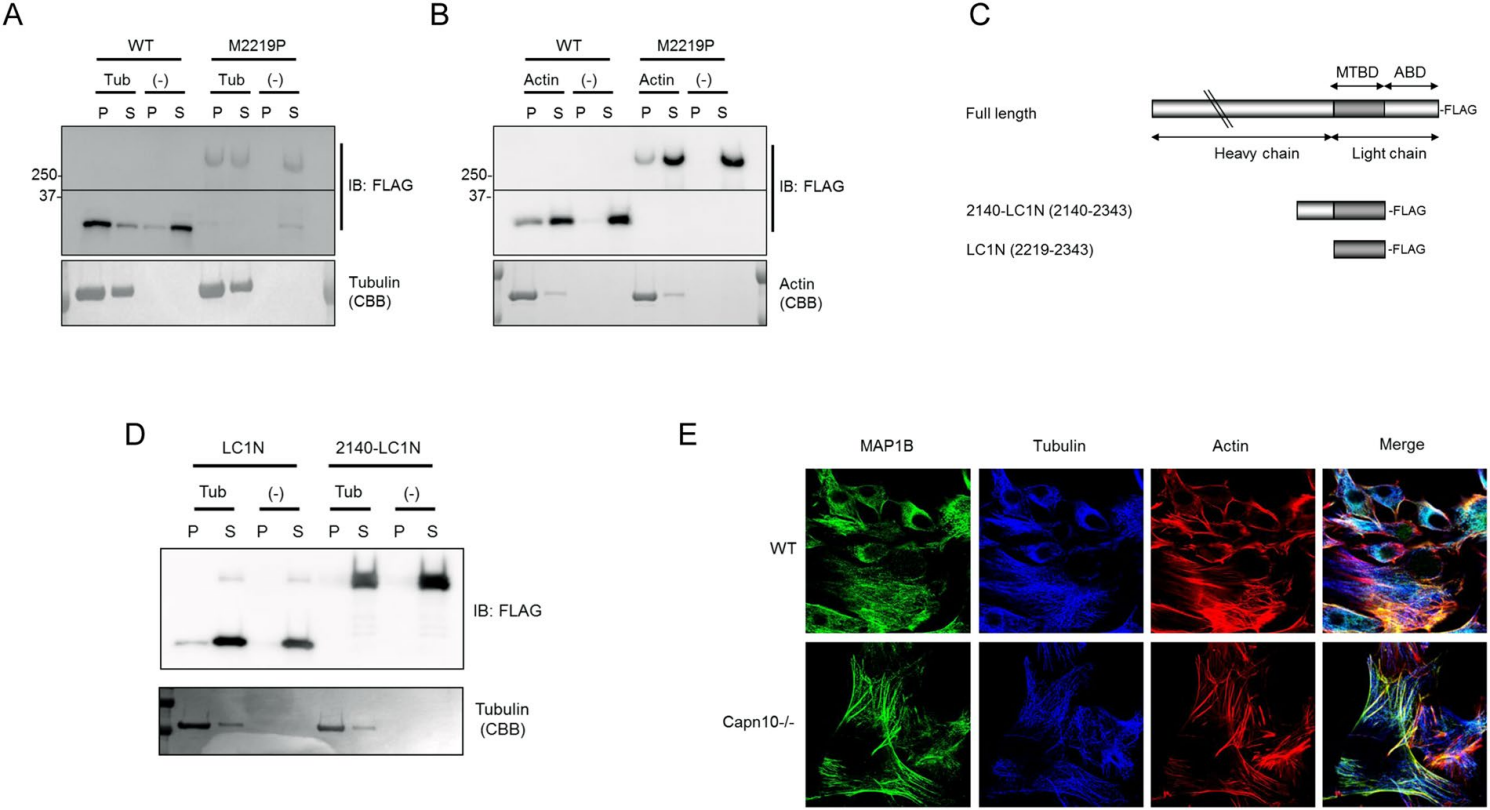
Microtubule binding activity of MAP1B activation by CAPN10. (**A**) Microtubule co-sedimentation assay *in vitro*. Microtubule binding ability of cleaved and uncleaved form of MAP1B was analyzed. Fully processed form of MAP1B (WT) and uncleaved MAP1B (M2219P) were sedimented in the presence (Tub) or absence (−) of polymerized microtubules. Equal amounts of precipitate (P) and supernatant (S) fraction were analyzed by western blotting probed with anti-FLAG antibodies. Tubulin was visualized by CBB staining. (**B**) Actin co-sedimentation assay *in vitro*. Actin binding ability of cleaved and uncleaved form of MAP1B was analyzed. Fully processed form of MAP1B (WT) and uncleaved full-length MAP1B (M2219P) were sedimented in the presence (Actin) or absence (−) of polymerized actin. Equal amounts of precipitate (P) and supernatant (S) fraction were analyzed by western blotting probed with anti-FLAG antibody. Actin was visualized by CBB staining. (**C**) Schematic representation of MAP1B full-length form, 2140-LC1N (2140–2343) and LC1N (2219–2343). (**D**) Microtubule binding ability of cleaved form of microtubule binding domain of the light chain (LC1N) and uncleaved form model of microtubule binding domain of the light chain (2140-LC1N) of MAP1B was analyzed by microtubule co-sedimentation assay *in vitro*. LC1N or 2140-LC1N was sedimented in the presence (Tub) or absence (−) of polymerized microtubules. Equal amounts of precipitate (P) and supernatant (S) fraction were analyzed by western blotting probed with anti-FLAG antibody. Tubulin was visualized by CBB staining. (**E**) Immuno-fluorescence micrographs of WT MEF cells (WT: upper panels) and Capn10^−/−^ MEF cells (Capn10^−/−^: lower panels) visualized with anti-MAP1B-Cterm (green: left panels), anti-tubulin (blue: middle panels) and TexasRed-phalloidin (red: right panels) are shown.

HCs and LCs are known to interact with each other and form mature complexes. LCs can bind microtubules by themselves, and HCs contain additional sequences that bind microtubules. In addition to microtubule binding activity, LCs contain an actin-binding domain. Thus, MAP1B has microtubule-stabilizing activity and is thought to be a microtubule-F actin-integrating protein. To better understand the function of CAPN10 processing, we characterized the microtubule-binding activity of full-length (M2219P) and processed MAP1B (WT) or HC/LC complex, since the microtubule-binding domain of LC is reported to be adjacent to the processing site of CAPN10^10^. The majority of HC/LC (WT) complex was co-precipitated with tubulin polymerized *in vitro* (Fig. 2A), as previously reported, whereas a considerable amount of M2219P remained in the supernatant fraction (Figs 2A and S6) in the co-sedimentation assay. This suggests that unprocessed MAP1B binds to microtubules more weakly than the mature HC/LC complex does, possibly because CAPN10 processing exposes and activates the microtubule-binding domain of LCs. It has been reported that MAP1B has two actin binding sites^10,11^, one located in the HC and the other in the LC. We also found that the actin-binding activity of M2219P was similar to WT, possibly because one actin binding site is located at the C-terminus of LCs and the other is located near the N-terminus of HCs (Figs 2B and S6). CAPN10 processing thus may not affect the actin binding activity of MAP1B. We then focused on the microtubule binding domain of the light chain, since it is located near the cleavage site. We examined whether the microtubule binding ability of the light chain differed when the light chain was cleaved from the heavy chain. We therefore designed MAP1B 2140–2343 (2140-LC1N) and 2219–2343 (LC1N) as an unprocessed and a cleaved form of microtubule binding domain MAP1B LC (Fig. 2C). These proteins were purified and their microtubule binding ability *in vitro* was determined as described above. LC1N bound to microtubules and was co-precipitated after centrifugation (Fig. 2D). In contrast, 2140-LC1N was not co-precipitated after centrifugation. This result suggests that 2140-LC1N could not bind to microtubules and indicates that the microtubule-binding domain became active and bound microtubules only when 2140-LC1N was cleaved and maturated.

### Full-length MAP1B dominantly binds to actin filaments and not to microtubules *in vivo*

Endogenous MAP1B was almost completely processed in WT MEF cells, but not in Capn10^−/−^ MEF, as shown in Fig. 1D. We therefore compared MAP1B localization in these two cell types. Confocal immunofluorescence microscopy showed thick actin stress fiber formation in Capn10^−/−^ MEF, and unprocessed MAP1B co-localized with these actin stress fibers (Fig. 2E). In WT MEF cells, MAP1B was co-localized with tubulin in accord with well-documented observation (Fig. 2E)^12^. These results are also consistent with the *in vitro* co-sedimentation assay described above. Together, the findings suggest that CAPN10 is an MAP1B processing enzyme necessary for the mature HC/LC complex formation required for localization to microtubules (Fig. S7).

### Calpain-10 knock-down impairs actin dynamics

**Figure 3:**
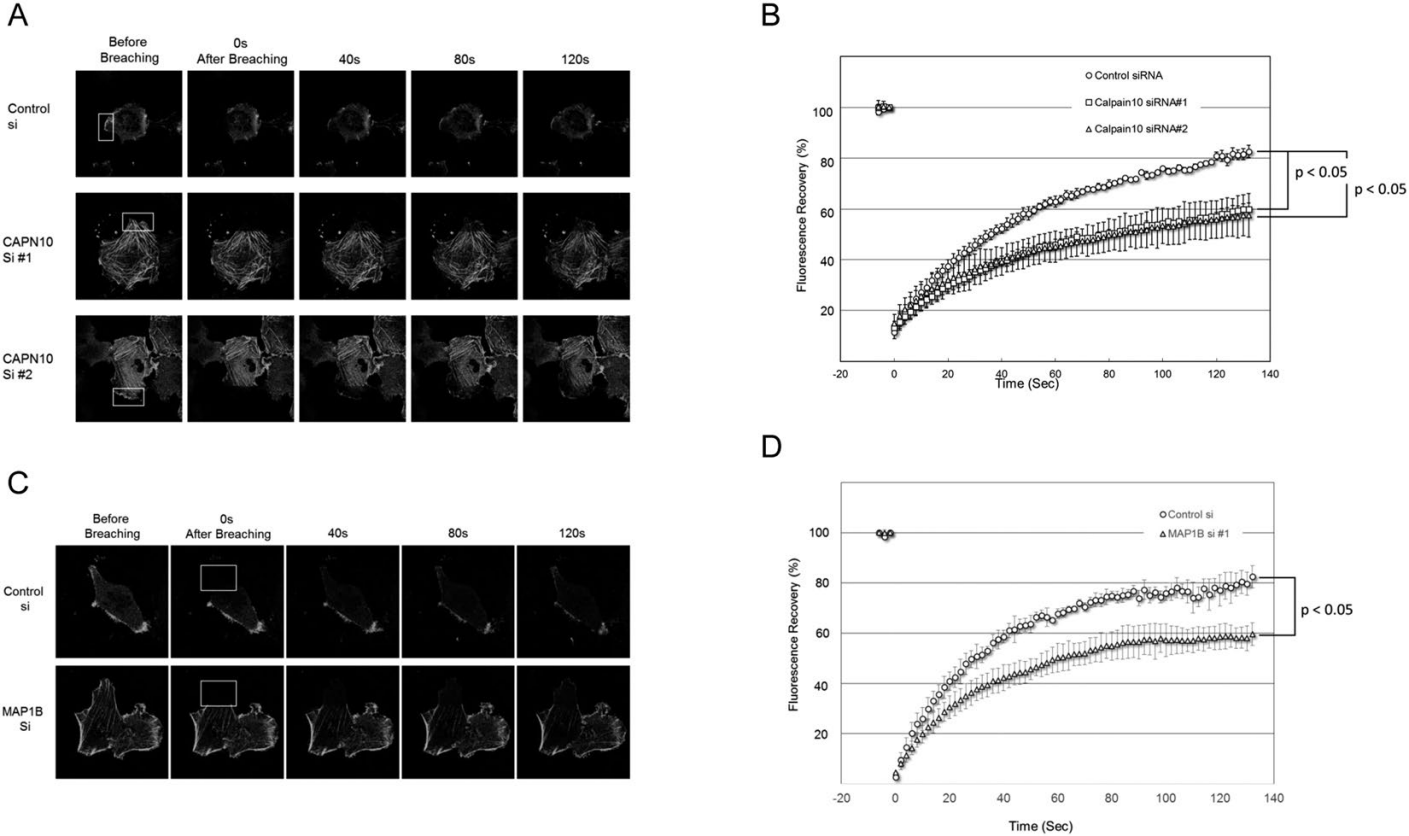
FRAP analysis of actin dynamics in HTC75 cells. (**A**) Representative images collected during FRAP experiment in HTC75 cells treated with control siRNA (upper panels) and CAPN10 siRNAs (middle and lower panels). Photobleached areas are indicated by boxes. (**B**) Percentages of fluorescent recovery are plotted for control siRNA (circles), CAPN10 siRNA#1 (squares), and CAPN10 siRNA#2 (triangles). Data show the mean values for 6 cells from a representative experiment. (**C**) Representative images collected during a FRAP experiment in HTC75 cells treated with control siRNA (upper panels) and MAP1B siRNA (lower panels). Photobleached areas are indicated by boxes. (**D**) Percentages of fluorescent recovery were plotted for control siRNA (circles), and MAP1B siRNA (triangles). Data show the mean values for 6 cells from a representative experiment.

We next examined actin dynamics in cells depleted of CAPN10 by siRNA knock-down method using the HTC75 cell line stably expressing GFP-actin (Fig. S8). As expected, CAPN10 siRNA treatment greatly increased the amount of thick stress fiber formation in HTC75, similar to Capn10^−/−^ MEF (Fig. 3A). To monitor actin dynamics of HTC75 cells, we photo-bleached actin stress fiber and measured fluorescence recovery after photo-bleaching (FRAP). FRAP analysis showed that CAPN10 siRNA significantly reduced the fluorescence recovery rate, whereas scrambled control siRNA had no such effect (Figs 3A,B and S9A,B). There is a statistically significant difference at every time point for si RNA#1 and for si RNA#2, after 22 sec and 38 sec, respectively (P < 0.05). We then examined actin dynamics in cells depleted of MAP1B by siRNA knock-down using the HTC75 cell line in a manner similar to that described above. As in the case of CAPN10, increased amounts of thick stress fiber formation and delayed fluorescence recovery were observed when the HTC75 cells were treated with MAP1B siRNA (Figs 3C,D and S9B). There is a statistically significant difference at every time point after 18 sec for MAP1Bsi RNA#1 (P < 0.05). These findings indicate that MAP1B itself also regulates actin dynamics. Considered together, the results of these CAPN10 and MAP1B knock-down experiments clearly indicate that CAPN10 regulates actin dynamics via MAP1B proteolytic processing.

### Glucose-stimulated insulin secretion in pancreatic islets of calpain-10 knockout mice

**Figure 4:**
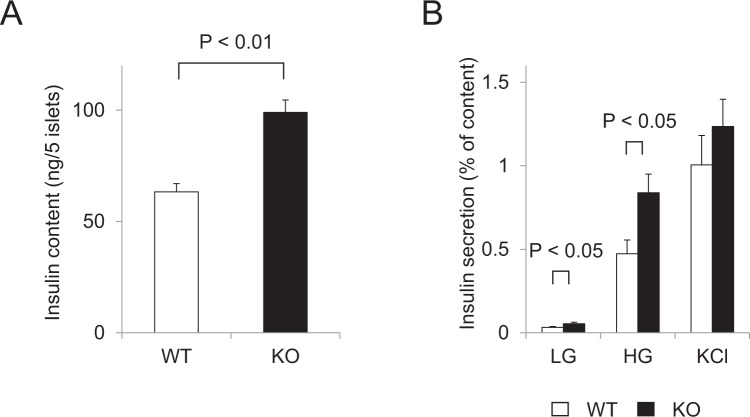
Glucose-stimulated insulin secretion in pancreatic islets of calpain-10 knockout mice. (**A**) Insulin content (**B**) insulin secretion under both low and high glucose levels, and KCl stimulation.

For pancreatic β-cells to secrete insulin in response to elevated blood glucose, insulin granules retained within the subplasmalemmal space must be transported to sites of secretion on the plasma membrane. It has been reported that microtubule-based insulin granule transport may act in concert with any actin-based transport that occurs within the subplasmalemmal space, rather than simply being a passive track for insulin granule delivery^13^. Calpain-10 regulates actin dynamics through MAP1B cleavage as described above. Therefore, we examined glucose-stimulated insulin secretion in pancreatic islets from calpain-10 knockout mice. As shown in Fig. 4A, insulin content of the islets of Capn10^−/−^ mice was increased 1.5-fold, compared to that of WT mice. Isolated islets of WT or Capn10^−/−^ mice were incubated under basal (2.8 mM Glucose) or stimulatory (16.7 mM Glucose or 60 mM KCl) conditions. Nevertheless, increased insulin release was observed not only under basal condition but also under glucose-stimulated condition with Capn10^−/−^ islets compared with WT islets (Fig. 4B). However, no significant difference in insulin release between WT and Capn10^−/−^ islets was observed under KCl stimulated condition. These results indicated that calpain-10 is associated with increased insulin contents and glucose-stimulated insulin release, but is not associated with KCl-stimulated insulin release (Fig. 4B).

## Discussion

In the present study, we identified MAP1 family proteins as the interacting and substrate proteins of CAPN10. It is known that MAP1 family proteins are synthesized as precursor proteins and that they are cleaved into heavy and light chains, but the processing protease has not been determined^9,14,15^. Our results demonstrate that CAPN10 processes the MAP1 family proteins into the heavy and the light chains both *in vivo* and *in vitro*. CAPN3 may also cleave MAPs at sites different from CAPN10, although CAPN3 is expressed only in skeletal muscle, and there is none detected in other tissues including heart muscle and smooth muscles such as intestine muscle. Further investigation is necessary to elucidate functional interaction between CAPN10 and CAPN3 with reference to vesicle transport such as GLUT4, etc.

It was reported that MAP1B is implicated in the coordinated assembly of cytoskeletal components required for branching and straight directional axon growth^16^. It also was reported that MAP1B-deficient neuronal cells show slow migration and late and abnormal morphology when treated with lysophosphatidic acid, an extracellular signaling phospholipid that triggers changes in actin distribution^17^. Therefore, it has been proposed that MAP1B coordinates microtubule and actin filament remodeling in neuronal cells. Considering these reports and our observations together, full-length MAP1B may well be able to impede actin dynamics and impair actin remodeling.

Previously, it was reported that targeted suppression of CAPN10 using RNA interfering protocols impaired insulin-stimulated glucose uptake in 3T3L1 adipocytes^3^ and primary human skeletal muscle cells^4^. However, suppression of CAPN10 expression was found to have no effects on the expression and insulin-stimulated phosphorylation of signaling molecules or glycogen synthesis in skeletal muscle cells^4^. Indeed, decreased CAPN10 expression was shown to decrease insulin-stimulated GLUT4 translocation in 3T3L1 adipocytes and to be associated with actin reorganization^3^.

Nevins *et al*., reported the effects of jasplakinolide treatment on insulin secretion of pancreatic beta-cells^18^. Jasplakinolide is known as an actin polymerization reagent that affects cortical actin rearrangement. Jasplakinolide treatment potentiated insulin secretion initiated by glucose, and slightly reduced insulin secretion initiated by KCl. On the other hand, somewhat different effects of Latrunculin B actin depolymerization reagent treatment on insulin secretion of the β cell has been reported. Latrunculin B treatment potentiated insulin secretion initiated by not only glucose but also KCl. Accordingly, it was thought that the abnormality of the actin dynamics caused by calpain-10 deficiency affects insulin secretion in a different manner from that of these drugs.

More recently, it was reported that homozygous mutations of CAPN10, an in frame insertion of five amino acids in the calpain catalytic domain and two kinds of nonsense mutation, were identified in patients with intellectual disability including cognitive disorders^19,20^. It has been speculated that there are common genetic factors linking diabetes and cognitive disorders such as Alzheimer’s disease, since the onset rate of this disease in patients with type 2 diabetes is two to three times higher than that in non-diabetic subjects. Indeed, Alzheimer’s disease may be considered as type 3 diabetes, as it exhibits brain-specific insulin resistance^21^. Impairment of MAP1B cleavage by abnormal CAPN10 might well cause cognitive disorders, as MAP1B has been shown to coordinate microtubule and actin filament remodeling in neural cells^17^.

Although the role of the CAPN10/MAP1B system is still not fully understood, numerous studies have implicated microtubules and F-actin formation in insulin secretion and insulin stimulated glucose uptake. Our study demonstrates that deficiency both of CAPN10 and MAP1B causes impairment of mircrotubule-F actin integration and leads to abnormal actin dynamics, which may affect insulin secretion. These findings clarify why CAPN10 was found to be a T2DM susceptibility gene and provide novel and intriguing insight into the pathophysiology of T2DM. These findings may also contribute to the development of novel drugs for treatment and prevention of the disease.

## Methods

### Cell culture and transfection

HEK293T, MEF and HTC75 cells were cultured in Dulbecco’s modified Eagle’s medium (DMEM) supplemented 10% FBS in 5% CO_2_ at 37 °C. Transfections of expression plasmids or siRNAs were performed with Lipofectamine 2000 or Lipofectamine RNAi MAX reagents (Life Technologies) according to the manufacturer’s instruction. The sequences of siRNAs are as follows;

CAPN10-HSS167081 (#1)

H01

ACAAGUCGCAGAAGAGCGAGGAGUC

H02

GACUCCUCGCUCUUCUGCGACUUGU

CAPN10-HSS167082 (#2)

H03

UAGCGAUGUGGCACGCAGCUCAGCA

H04

UGCUGAGCUGCGUGCCACAUCGCUA

CAPN10-HSS167083 (#3)

H05

UGGAUGGCCUGUCAAUCCUGGUUGC

H06

GCAACCAGGAUUGACAGGCCAUCCA

MAP1B-HSS106327 (#1)

F9

UUCAAUAGGAGACUCUGAGUCAGGC

F10

GCCUGACUCAGAGUCUCCUAUUGAA

### Plasmid construction

All plasmids used in this study were constructed using GateWay system (Thermo fisher scientific) according to the manufacturer’s instructions. FLAG tagged expression vector was prepared by using pDEST12.2-3′ FLAG plasmid, which has a FLAG sequence at the 3′ end of the recombination site as a destination vector. GST tagged expression vector was prepared by using pDEST26 plasmid as a destination vector.

### Protein identification

FLAG-tagged calpain-10 was expressed in HEK293T cells and immunoprecipitated using FLAG M2 beads (Sigma-Aldrich). Interacting proteins co-immunoprecipitated with calpain-10 were eluted with FLAG peptide (Peptide Inst.) and were analyzed as described as previously^8^.

### Western blotting analysis

Cells were lysed in lysis buffer (20 mM Tris pH 7.5, 0.5% Triton X-100, 150 mM NaCl, phosphatase inhibitor mix (Roche), protease inhibitor mix (Roche)) on ice and clarified by centrifugation. Cell extracts were resolved on SDS-PAGE and transferred onto PVDF membrane. After blocking with 5% skim milk in TBS-T (20 mM Tris pH 7.5, 150 mM NaCl, and 0.1% Tween20), the membranes were probed with antibodies. Anti-FLAG M2 (Sigma-Aldrich), anti-tubulin (Sigma-Aldrich), anti-MAP1B C-terminus (H130; Santa Cruz Biotechnology) and anti-GST-HRP (GE healthcare) were used as probes and detection was performed using the ECL plus detection kit (GE healthcare).

### *In vitro* digestion and N-terminal sequencing

MAP1B-FLAG protein was partially purified from transiently transfected HEK293T cells. MAP1B-FLAG protein was immunoprecipitated with FLAG M2 beads (Sigma-Aldrich) and eluted with FLAG peptide. GST-CAPN10_WT and _C73S were also purified from transiently transfected HEK293T cells using glutathione sepharose beads (GE healthcare) (Fig. S3). Each eluted protein was incubated in reaction buffer (20 mM Tris pH 7.5, 150 mM NaCl, 5 mM DTT) containing 2 mM CaCl_2_ or 2 mM EGTA for 4 hours at 37 °C. Reactions were stopped by the addition of 2× SDS sample buffer and were separated by SDS-PAGE and analyzed by western blotting probed by anti-FLAG and anti-GST antibody. N-terminal sequencing sample was prepared using the same protocol described above in the presence of CaCl_2_. Transferred membrane was visualized with CBB staining and an increased band was subjected to N-terminal amino acid sequence analyzer (*Applied Biosystems*).

### CAPN10 KO MEF construction

Generation of Capn10^−/−^ mice with targeted deletion of exon 1 and 2 of the Capn10 locus were generated through standard gene knockout technology. The exons 1 and 2 of Capn10 were replaced with a selective cassette containing a neomycin-resistance gene and a lacZ gene with a nuclear localization signal. The Capn10^+/−^ mice were then backcrossed to C57BL/6J for at least six generations for further studies. Mouse Embryonic Fibroblast (MEF) Timed pregnancy of heterozygous Capn10^+/−^ mating was used to generate MEFs. MEFs were isolated from Capn10^+/+^ and Capn10^−/−^ embryos at 13.5 days post coitum. Cells were cultured in Dulbecco¹s modified Eagle¹s medium (DMEM) supplemented with 10% FBS. Cells from passages 4 through 6 were used in experiments.

### Microtubule and actin co-sedimentation analysis

Purified tubulin (Cytoskelton) was dissolved and polymerized in TBS buffer (20 mM Tris-HCl pH 7.4, 150 mM NaCl, 2 mM EGTA and 1 mM MgCl_2_) containing 2 mM DTT, 1 mM GTP and 50 µM paclitaxel and incubated at 37 °C for 30 min. FLAG tagged MAP1B and FLAG tagged MAP1B-M2219P were produced in HEK293T cells by co-transfection with CAPN10 or CAPN10-C73S to produce cleaved form and uncleaved full-length MAP1B, respectively. Purified proteins were centrifuged at 100,000 × g for 30 min at 37 °C. Supernatants were added to polymerized tubulin and incubated for 30 min at 37 °C and centrifuged at 20,000 × g for 30 min at 37 °C. The pellets and supernatants were analyzed by western blotting probed with anti-FLAG antibody. For co-sedimentation analysis of the microtubule binding domain of MAP1B light chain, purified tubulin (Cytoskelton) was dissolved and polymerized in PEM buffer (100 mM Pipes pH 6.9, 2 mM EGTA, 1 mM MgCl_2_ and 2 mM DTT) containing 1 mM GTP and 50 µM paclitaxel and incubated at 37 °C for 30 min. FLAG tagged LC1N (2219–2343) was produced in HEK293T cells by co-transfection with FLAG tagged 2140-LC1N with CAPN10 for cleavage at 2218–2219 bond *in vivo*. FLAG tagged 2140-LC1N was produced in HEK293T cells by co-transfection with CAPN10-C73S. Purified LC1N and 2140-LC1N were dialyzed against PEM buffer and the same procedure was performed as described above.

### Immunofluorescence microscopy

MEF cells were cultured in a poly-L-lysine coated glass bottom dish and treated with 20 mM Hepes pH 7.5, 10 mM KCl, 1.5 mM MgCl_2_, 1 mM EGTA, 1 mM EDTA and 250 mM sucrose containing 50 µg/ml digitonin for 5 min. Cells were fixed with 4% PFA in 60 mM Pipes, 25 mM Hepes, 10 mM EGTA and 2 mM MgCl_2_ for 30 min. MEF cells were blocked with 2% BSA in PBS. Immunostaining was performed using mouse anti-tubulin antibody (Sigma-Aldrich) and rabbit anti-MAP1B C-terminus (H130; Santa Cruz Biotechnology) for 1 hour, followed by treatment with anti-mouse IgG Cy-5 conjugate, anti-rabbit IgG Alexa Fluor 488 conjugate and TexasRed-phalloidine (Molecular Probes). Samples were examined with confocal laser microscope (Olympus).

### FRAP assay

HTC75 cells stably expressing GFP-actin cultured in DMEM with 10% FBS were transfected with siRNA for control, CAPN10 or MAP1B using the RNAiMAX transfection reagent (Invitrogen) and cultured for 48–72 hr before analysis. For FRAP (fluorescence recovery after photobleaching), siRNA-treated cells were incubated in HEPES-buffered DMEM and placed on a microscope stage that had been pre-warmed at 37 °C. The cells were observed and bleached using a FV1000 confocal microscopy system (Olympus). To visualize FRAP of GFP-actin, images were acquired sequentially every 2 sec. All values are indicated as mean ± standard deviation (SD). The unpaired student-*t* test was used for statistical analysis. Statistical analysis was performed with JMP® version 11 software (SAS Institute, Inc., Cary, NC).

### Glucose-stimulated insulin secretion in pancreatic islets of calpain-10 knockout mice

As described previously^22^, pancreatic islets were isolated from WT and calpain-10 KO mice by collagenase digestion and cultured for 2 days. Thirty minutes after preincubation of isolated islets with HEPES-KRB buffer containing 2.8 mM glucose, five size-matched islets were collected in each well of a 96-well plate and incubated for 30 min in 100 μl of the same buffer containing various stimuli. Insulin released in the incubation buffer and cellular insulin content were measured by ELISA (Medical Biological Laboratories, Nagoya, Japan). The amount of insulin secretion was normalized by cellular insulin content.

Animal experiments were carried out in accordance with the National Institutes of Health Guide for the Care and Use of Laboratory Animals (NIH Publication No. 8023, revised 1978). All animal care was approved by the Animal Care Committee of Gifu University (No. 27–37).

## Electronic supplementary material

Supplementary Information
