# Supplementary material for: Calpain-10 regulates actin dynamics by proteolysis of microtubule-associated protein 1B
Source: Sci Rep. 2018 Nov 13;8:16756. doi: 10.1038/s41598-018-35204-x (PMC6233169; doi:10.1038/s41598-018-35204-x)

Supplemental Information

Calpain-10 regulates actin dynamics by proteolysis of microtubule-associated protein 1B

Tomohisa Hatta, Shun-ichiro Iemura, Tomokazu Ohishi, Hiroshi Nakayama, Hiroyuki Seimiya, Takao Yasuda, Katsumi Iizuka, Mitsunori Fukuda, Jun Takeda, Tohru Natsume and Yukio Horikawa

Supplementary Figure legends

Figure S1. Western blot analysis of cell lysates expressed the FLAG tagged calpain family with MAP1B-(Myc) x6 (A), MAP1A-(Myc) x6 (B) or MAP1S-(Myc) x6 (C). All proteins were transiently expressed in HEK293T cells and probed with anti-Myc and anti-FLAG antibody. Full length, cleavage product bands are indicated by arrows and arrowheads, respectively.

Figure S2. Western blot analysis of cell lysates from HEK293T cells transiently transfected with control plasmid (-), calpain-10-WT-FLAG (WT) or calpain-10-C73S-FLAG (C73S) (A), calpain-10 siRNA (CAPN10 si #1, CAPN10 si #2 and CAPN10 si #3) or control siRNA (control si) (B) and cell lysates from wild-type (WT) and calpain-10 knock-out (Capn10-/-) mouse embryonic fibroblasts (C) were analyzed by western blotting and probed with anti-MAP1S antibody. Full length, cleavage product bands are indicated by arrows and arrowheads, respectively. (D) Western blot analysis of Capn10-/- mouse brain lysate probed with anti-MAP1B_C-term (C20) recognizing both of MAP1B and MAP1A.

Figure S3. CBB stained SDS-PAGE images of partially purified GST-CAPN10 and

MAP1B-FLAG proteins used for *in vitro* digestion assay.

Figure S4. CBB stained SDS-PAGE image of *in vitro* digested MAP1B protein used for N-terminal sequencing analysis.

Figure S5. (A) Amino acid sequences near the cleavage site of human MAP1 family proteins were aligned by ClustalX program. The cleavage site of MAP1B determined in this study is indicated by arrow. Approximate site of cleavage inferred from previous work (Hammarback et al, 1991) is indicated. Dark and light shaded boxes highlight proline and charged residues. Conserved amino acids among the MAP1 family are marked by asterisk (completely conserved), colon (highly conserved) or period (partially conserved). (B), (C) Western blot analysis of CAPN10 resistant mutant of MAP1A (B) and MAP1S (C). Full length and cleavage product bands are indicated by arrows, and arrowheads, respectively.

Figure S6. Uncropped western blot images for Figure 2A and 2B.

Figure S7. Confirmation of MAP1B HC/LC dimer complex formation cleaved by

CAPN10. Western blot analysis of cell lysates (input) and immunoprecipitants using FLAG M2 antibody (elution) from HEK293T cells transfected with MAP1B_WT-FLAG (WT) or MAP1B_M2219P-FLAG (M2219P) and GST-CAPN10. MAP1B_HC and _LC (C-terminus) were probed with anti-MAP1B_HC and anti-FLAG antibody, respectively.

Figure S8. Confirmation of knock down efficiencies of CAPN10 siRNAs for HEK293 cells by qPCR. Triplicated samples were prepared for each siRNA and analyzed. The mRNA amounts of CAPN10 of each sample was plotted as relative units to the control siRNA treated sample.

Figure S9. (A) Confirmation of knock down efficiencies of CAPN10 siRNAs for HTC75 cells by qPCR. Triplicated samples were prepared for each siRNA and analyzed. The mRNA amounts of CAPN10 of each sample was plotted as relative units to the control siRNA treated sample. (B) Western blot analysis of cell lysates from HTC75 cells treated with calpain-10 siRNA (CAPN10 si #1 and CAPN10 si #2) or MAP1B siRNA (#1) probed with anti-MAP1B_LC1 antibody.

Figure S10. Fluorescence microscopy images of HTC75 cells stably expressed GFP-actin

treated with control siRNA, CAPN10 siRNA (#1), MAP1B siRNA (#1) or CAPN10 siRNA (#1)/ MAP1B siRNA (#1).

Supplemental methods

Plasmid construction

Plasmid construction were carried out in the same procedure as described in main text. For myc-tagged construct, we used pDEST12.2-3’(Myc) x6 plasmid as a destination vector.

Immunoprecipitation

Transfected HEK293T cells were lysed in a lysis buffer (20 mM Tris-HCl, pH 7.5, 150 mM NaCl, 0.5% Triton X-100, 1 mM Na_3VO_4, 50 mM NaF and protease inhibitor cocktail [complete EDTA-free protease inhibitor; Roche]). After centrifugation at 20000 g for 10 min, the supernatants were subjected to immunoprecipitation using anti-FLAG M2 beads (Sigma-Aldrich). Precipitated immunocomplexes were washed three times in washing buffer (10 mM Tris-HCl, pH 7.5, 150 mM NaCl and 0.1% Triton X-100) and eluted with FLAG peptide. Eluted samples were added SDS sample buffer and subjected to western blot analysis.

RNA Extraction and qPCR

siRNA treated cells were lysed, and RNA extraction was performed using RNeasy RNA

purification kit (Qiagen). The quality and purity of RNAs were determined by UV spectrometry. To obtain cDNA, we subjected 1μg total RNA to reverse transcription. The primer sequences used in this study were as follows: CAPN10: 5'-AGTTTGACGAGCTCACCGTT-3' (Forward), 5'-AGGACGGCAATGTACACCTC-3' (reverse); and internal control actin: 5'-TGGATCAGCAAGCAGGAGTATG-3' (forward), 5'-GCATTTGCGGTGGACGAT-3' (reverse). The reaction conditions of qPCR were the following: one cycle at 95°C for 10 min for initiation; 40 cycles at 95°C for 3 sec and 60°C for 30 s for extension. All the reactions were carried out in triplicate on a Step One Plus real-time PCR system (Thermo Fisher Scientific).

Supplemental figure S1

A

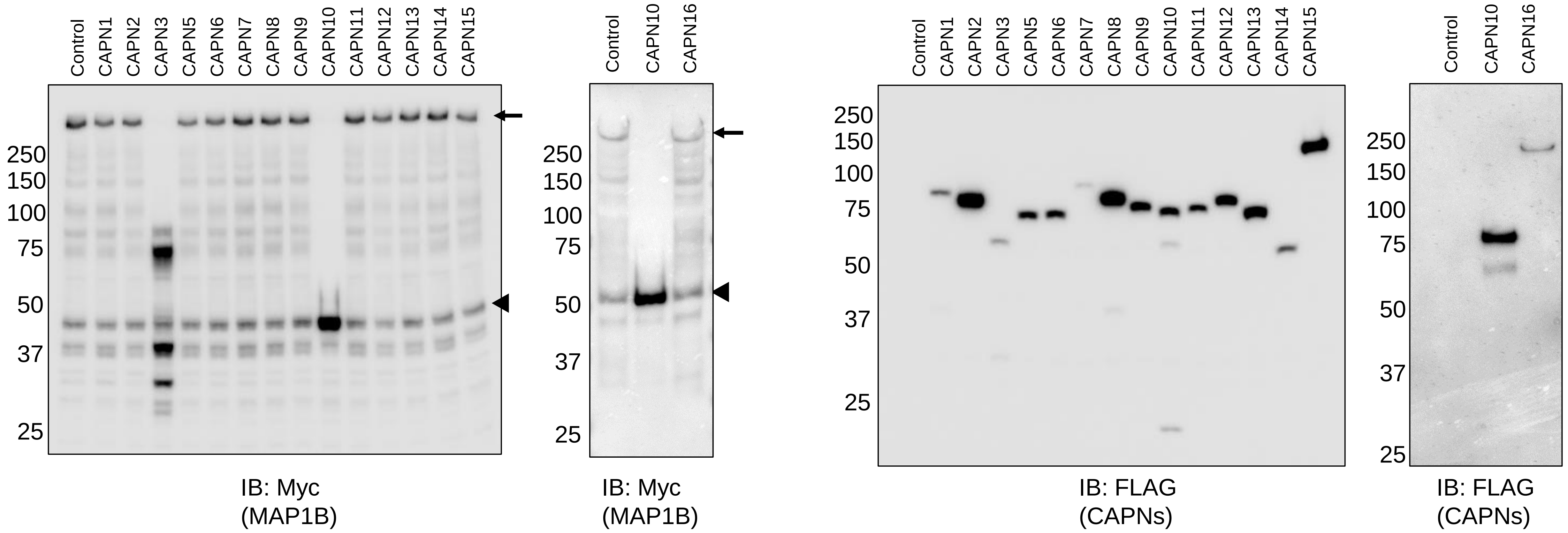

Supplemental figure S1

B

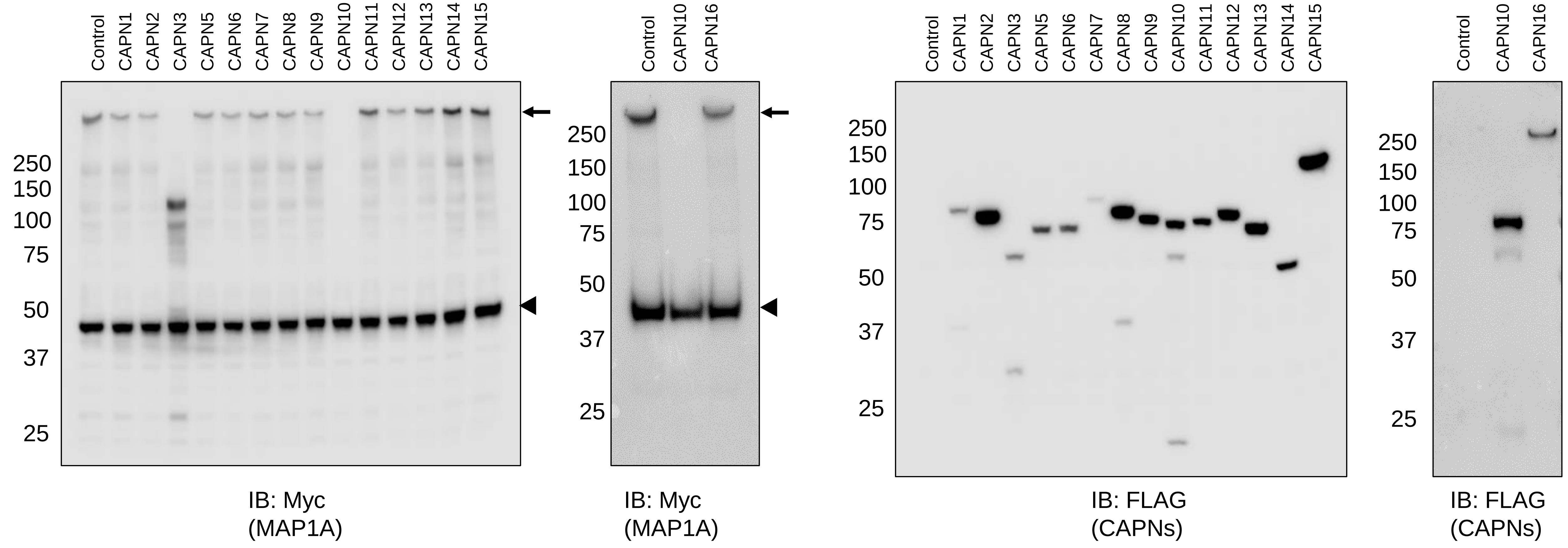

Supplemental figure S1

C

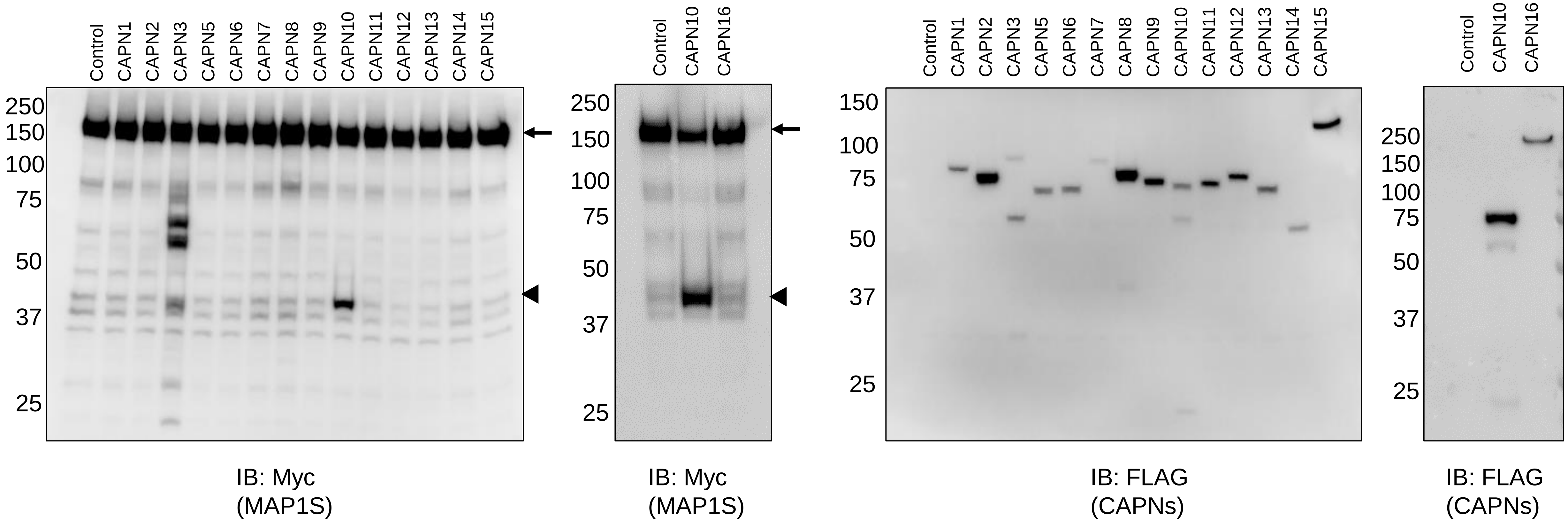

Supplementary Figure S2

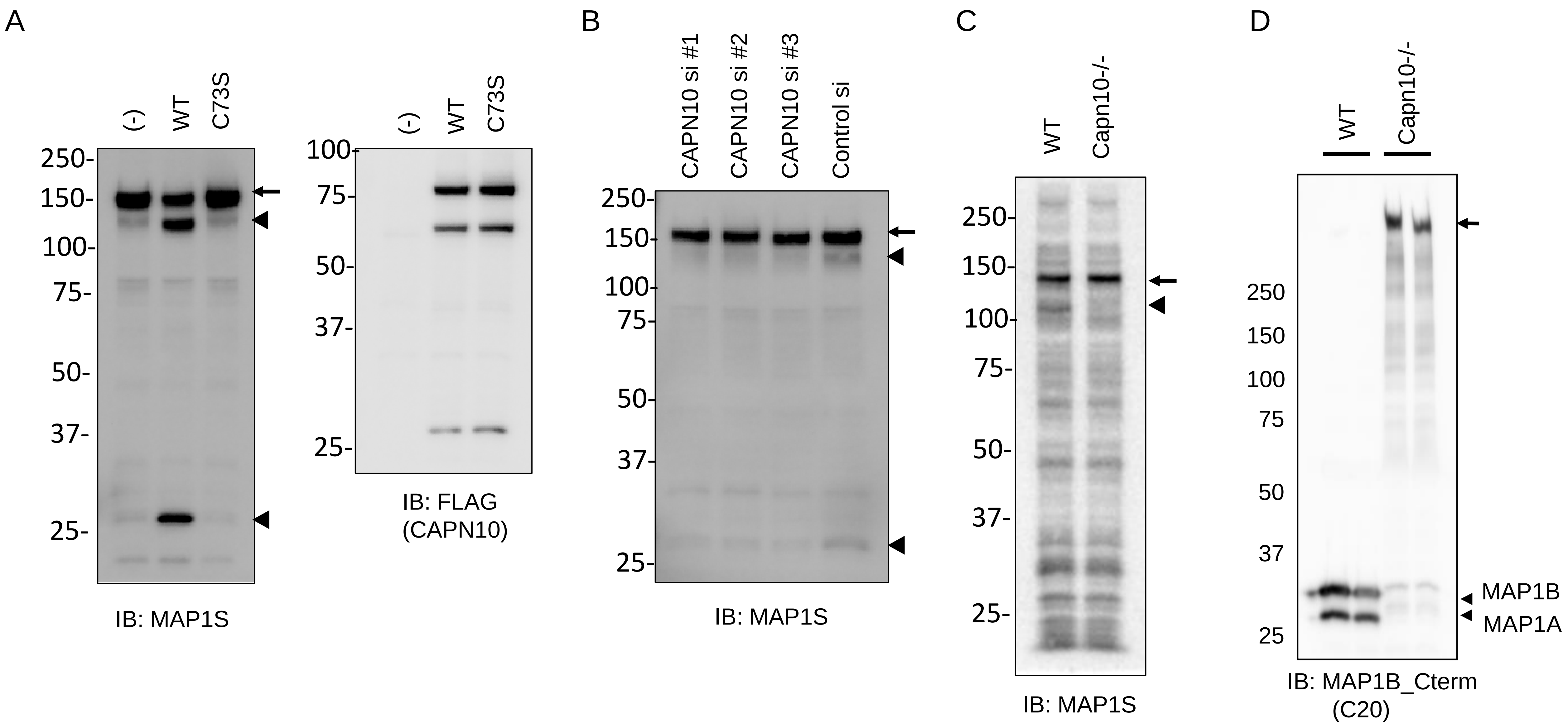

Supplemental figure S3

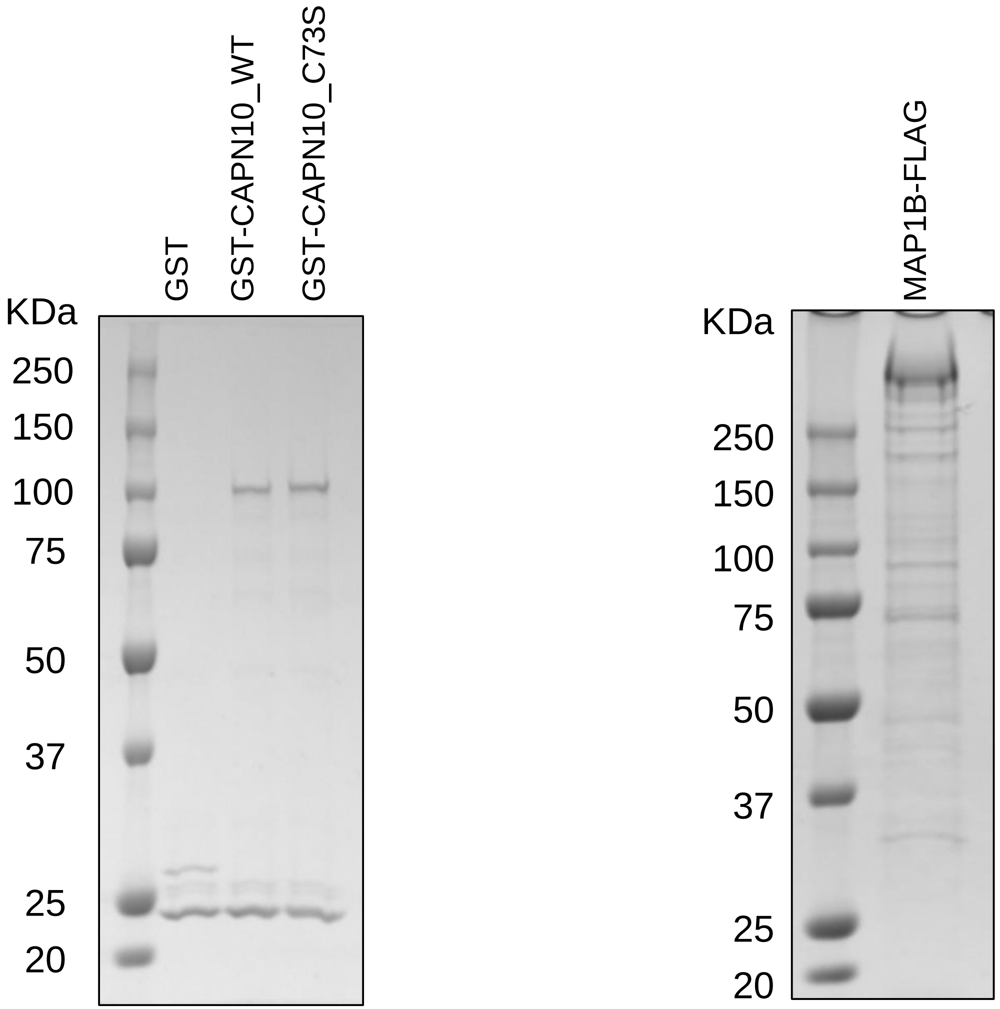

Supplemental figure S4

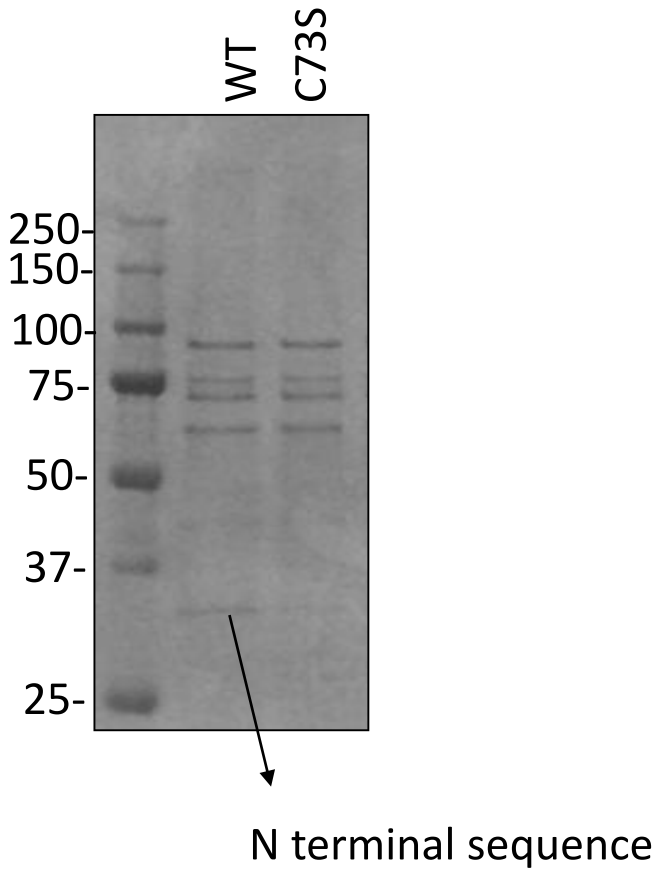

Supplementary Figure S5

A

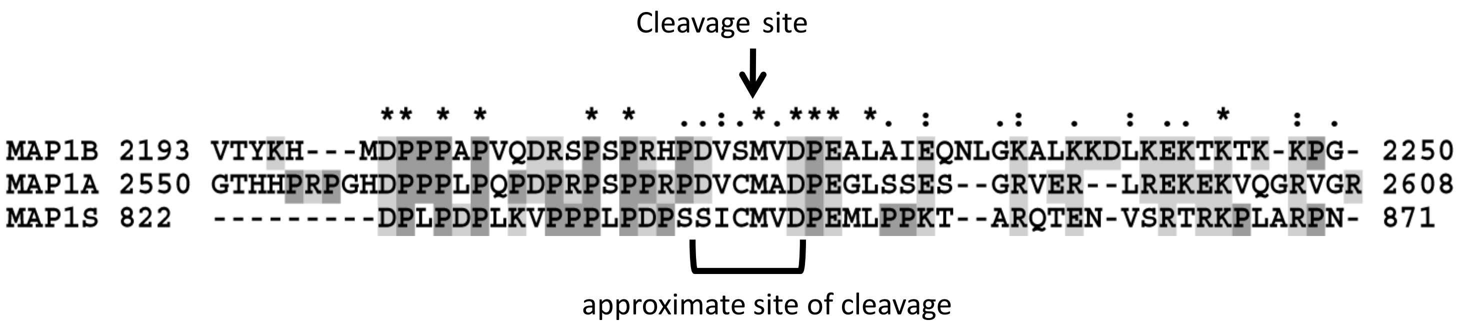

Supplementary Figure S5

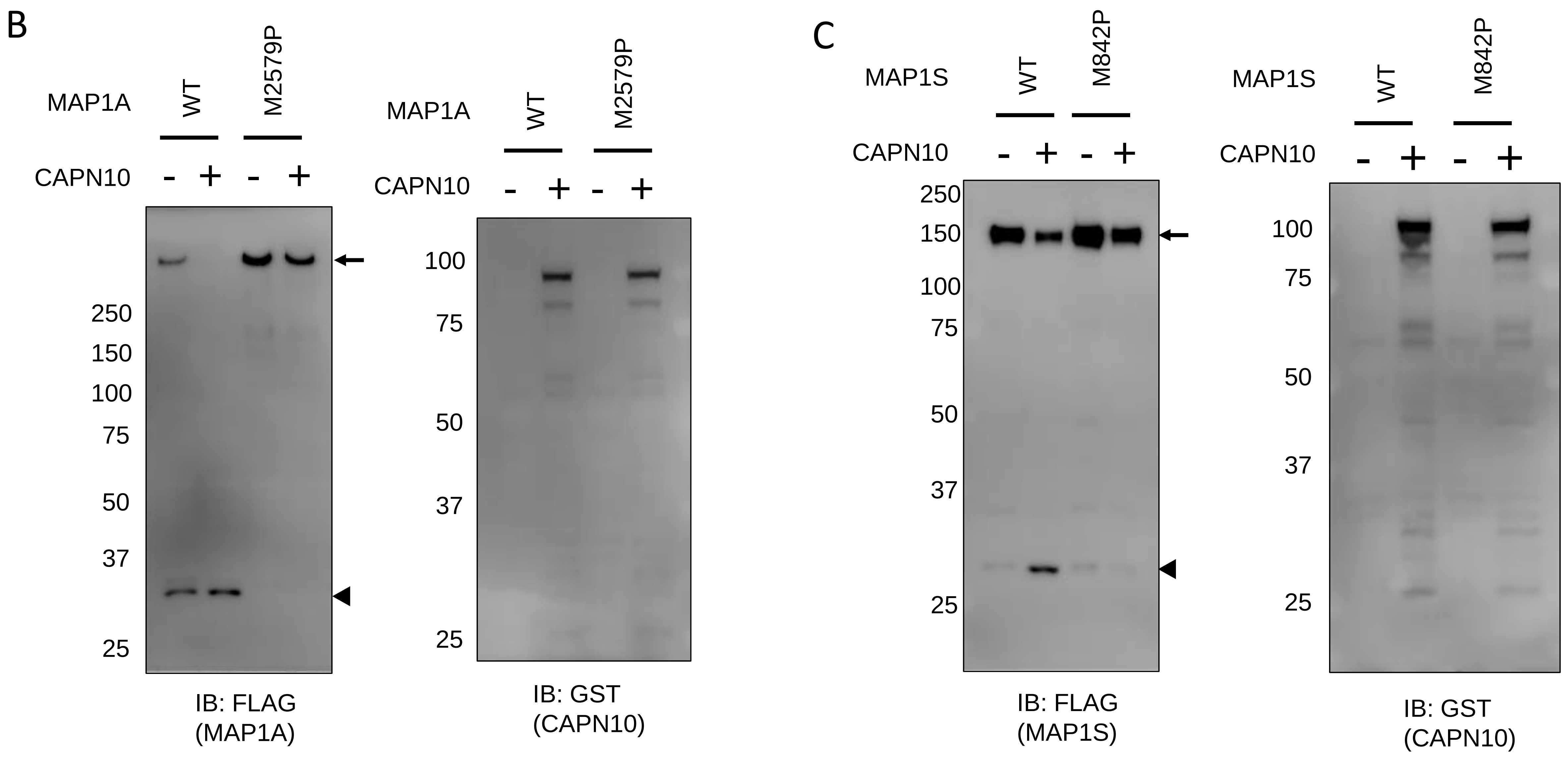

Supplementary Figure S6

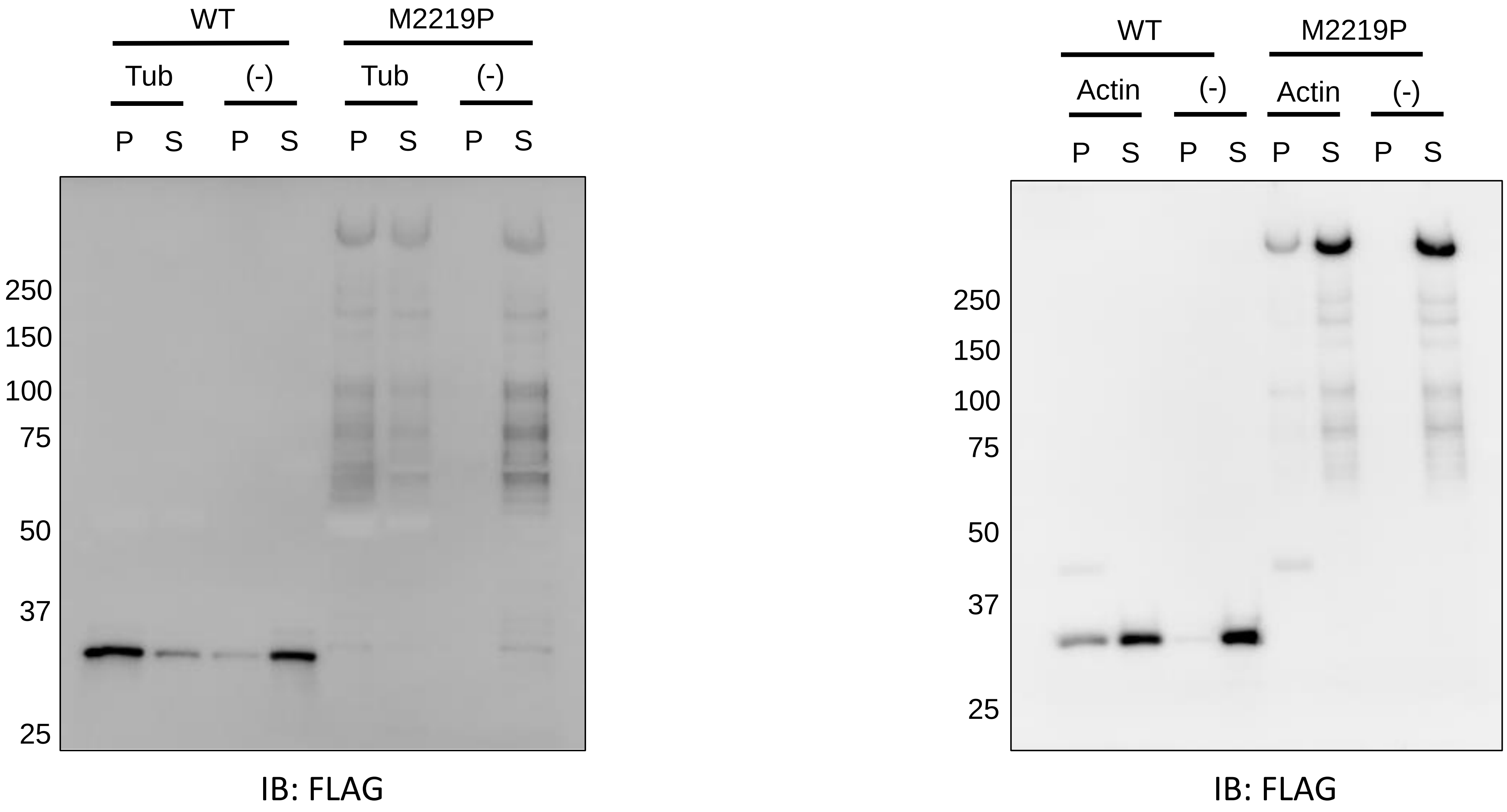

Supplementary Figure S7

HC/LC complex

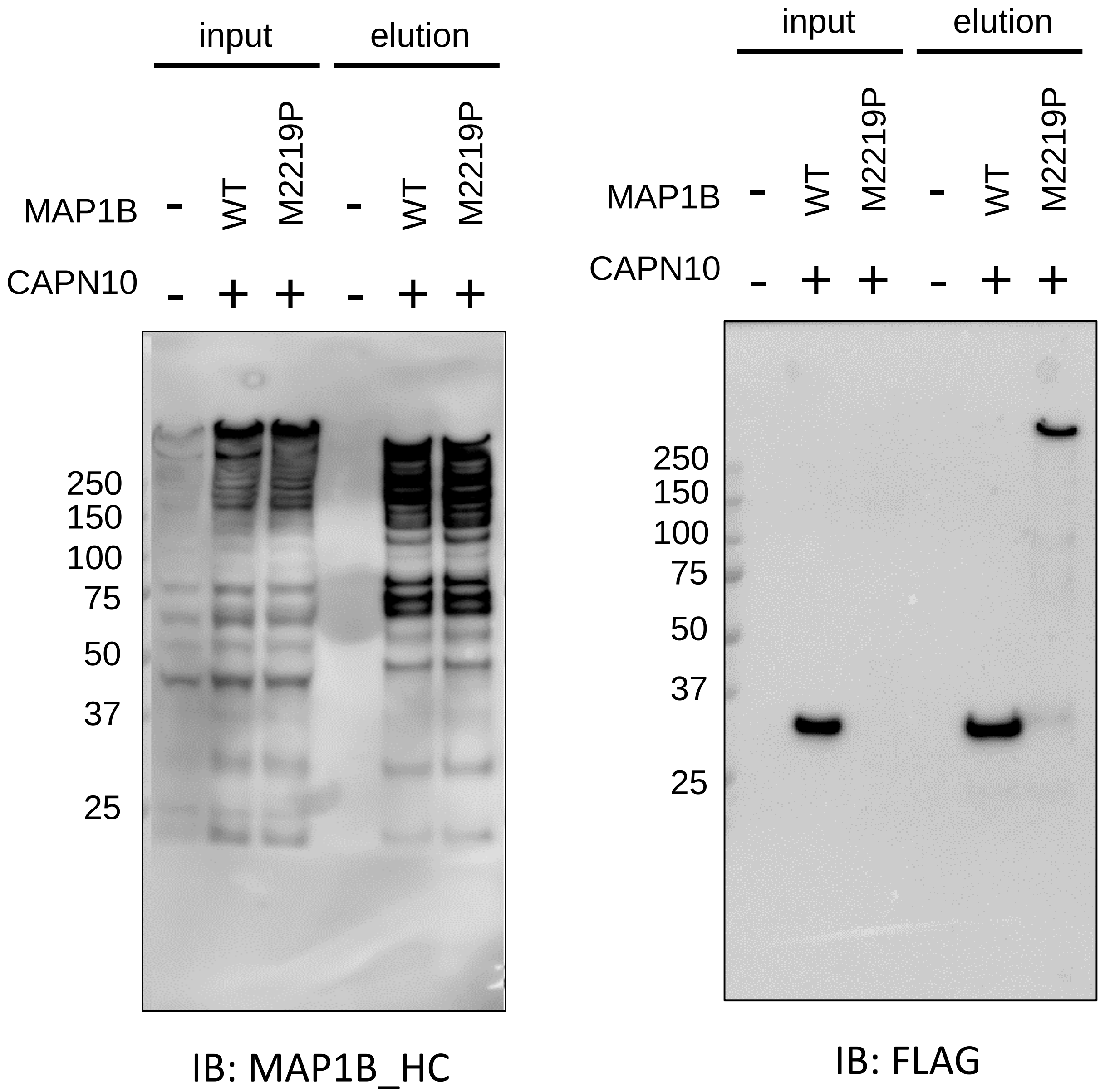

Supplemental figure S8

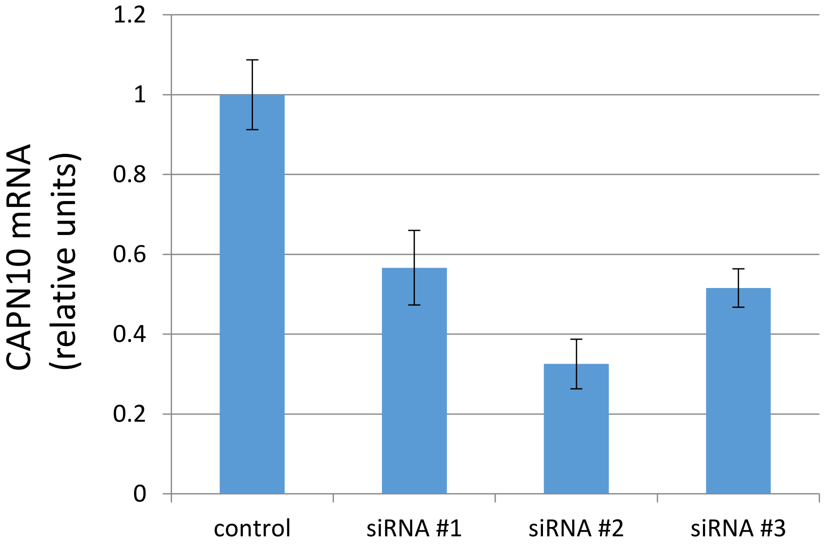

Supplementary Figure S9

A

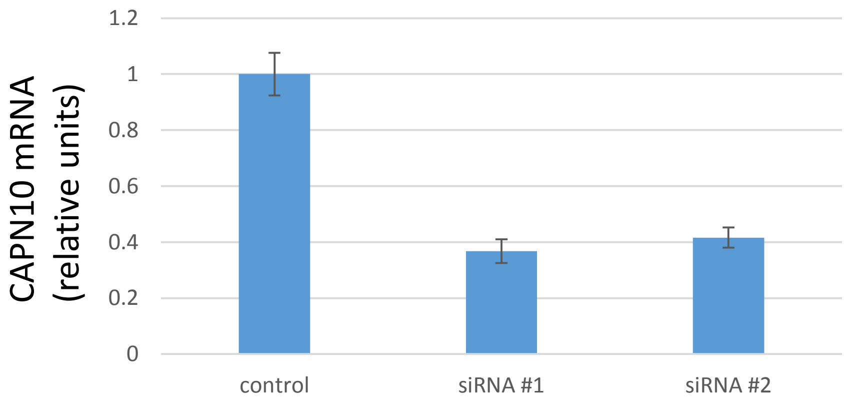

B

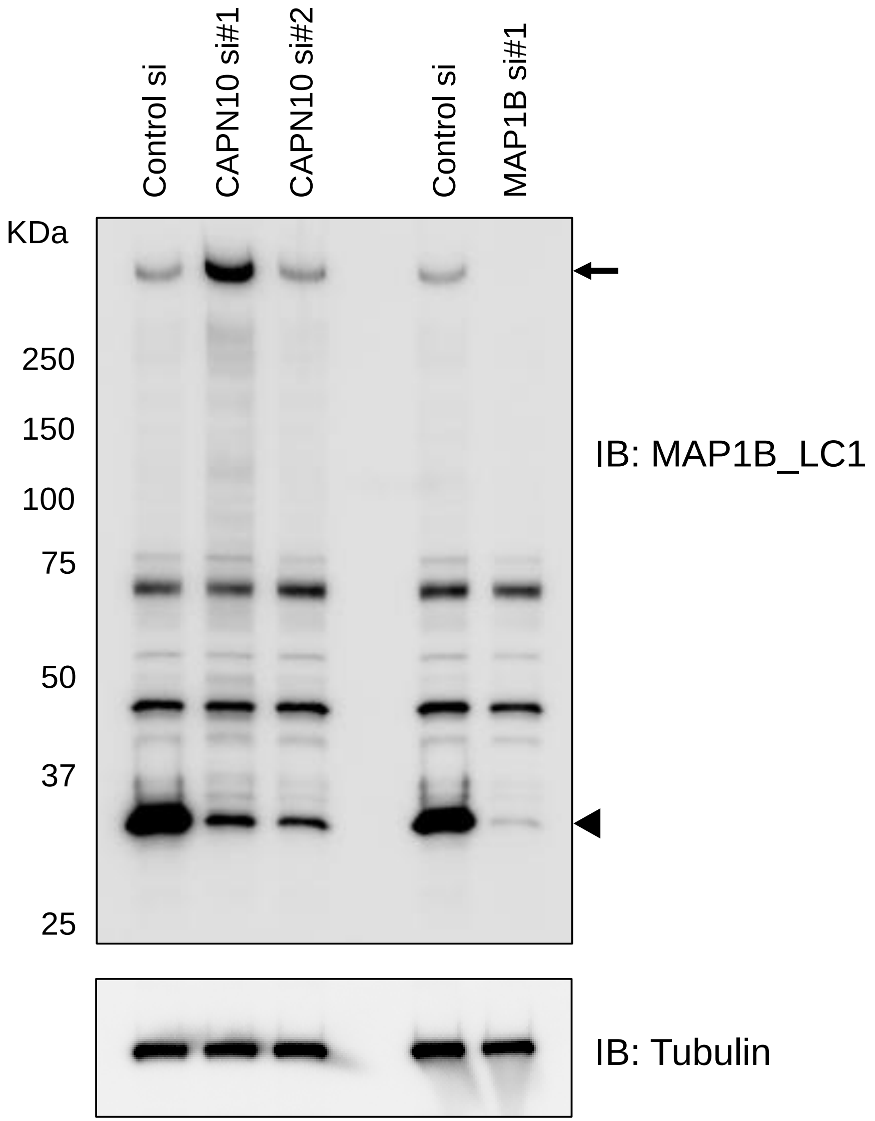

Supplementary Figure S10

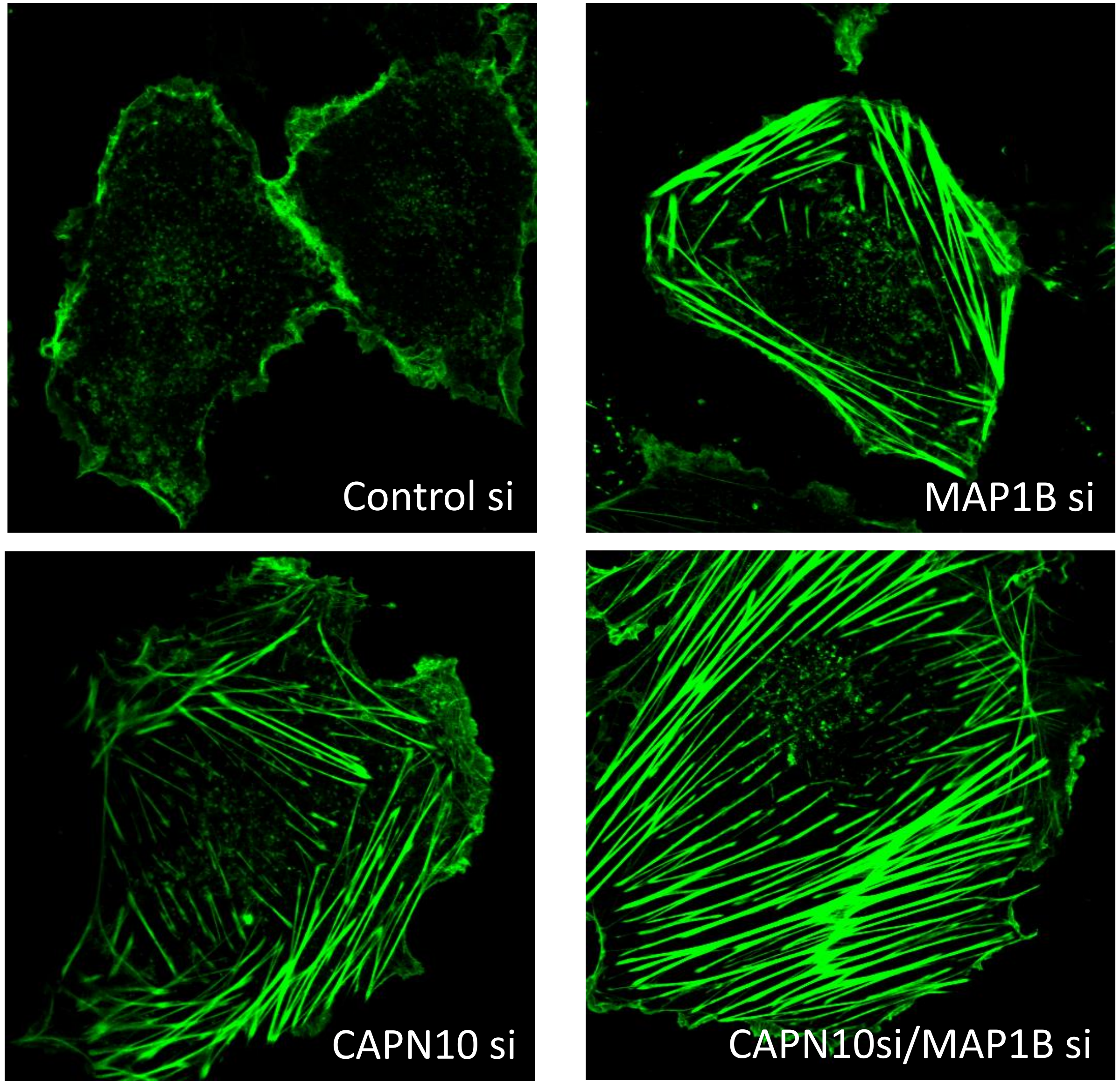

Supplement: Supplementary file 1 — Supplementary Information [file 41598_2018_35204_MOESM1_ESM.pdf]
